# AI-Assisted Preoperative Diagnosis of Wilms Tumor

**DOI:** 10.3390/life16040659

**Published:** 2026-04-13

**Authors:** Mustafa Alper Akay, Ozan Can Tatar, Elif Tatar, Uğur Demirsoy, Yonca Anık, Gülşen Ekingen Yıldız, Onursal Varlıklı

**Affiliations:** 1 Department of Pediatric Surgery, School of Medicine, Kocaeli University, İzmit 41001, Türkiye; pedcerr@gmail.com (M.A.A.); yukselelif77@gmail.com (E.T.); gulsen.ekingen@kocaeli.edu.tr (G.E.Y.); ovarlikli@gmail.com (O.V.); 2 Department of General Surgery, Kocaeli Sehir Hastanesi, İzmit 41060, Türkiye; 3 Department of Pediatrics, School of Medicine, Kocaeli University, İzmit 41001, Türkiye; ugur.demirsoy@kocaeli.edu.tr; 4 Department of Radiology, School of Medicine, Kocaeli University, İzmit 41001, Türkiye; yonca.anik@kocaeli.edu.tr

**Keywords:** deep learning, Wilms tumor, pediatric renal cancer, neuroblastoma, AI-assisted imaging

## Abstract

Preoperative differentiation of Wilms tumor and neuroblastoma on pediatric abdominal computed tomography (CT) images may be challenging because of overlapping imaging features. We aimed to develop an artificial intelligence-assisted lesion-localization model for exploratory diagnostic support in this differential setting. In this single-center, retrospective, image-level study, a YOLO26s detector was trained on preoperative contrast-enhanced CT PNG images with histopathology-anchored labels. The dataset comprised 3553 images, including 2103 lesion-positive images and 1450 background-negative images, partitioned into training, validation, and test subsets. On the held-out test set, the model achieved a precision of 0.954, a recall of 0.951, an mAP@0.5 of 0.977, and an mAP@0.5:0.95 of 0.732. Class-specific mAP@0.5:0.95 values were 0.734 for neuroblastoma and 0.730 for Wilms tumor. At the image level, tumor-present versus background-negative discrimination yielded 99.5% sensitivity, 89.0% specificity, a 93.0% positive predictive value, a 99.2% negative predictive value, and 95.3% accuracy. YOLO26s showed strong within-dataset performance for lesion localization and differential support between Wilms tumor and neuroblastoma.

## 1. Introduction

Wilms tumor (WT), a major pediatric renal malignancy, presents a substantial diagnostic challenge due to its heterogeneous imaging appearance and close resemblance to other renal lesions [1,2,3,4]. As the most common primary renal cancer in children, WT accounts for the majority of pediatric renal malignancies and requires timely recognition to ensure appropriate risk-adapted management. However, pediatric renal masses encompass a broad pathological spectrum—including clear cell sarcoma, rhabdoid tumors, congenital mesoblastic nephroma, inflammatory conditions, and other rare entities—that may radiologically mimic WT [1,2,3,4]. This overlap complicates initial evaluation and increases the risk of diagnostic uncertainty.

Accurate preoperative diagnosis is critical, as misclassification may lead to inappropriate treatment strategies and adversely affect clinical outcomes. Contemporary treatment protocols in pediatric oncology rely on precise tumor identification to guide decisions regarding upfront surgery, neoadjuvant chemotherapy, or biopsy. Inaccurate radiological interpretation may result in the overtreatment of benign lesions, delayed intervention for malignant tumors, or suboptimal surgical planning. The diagnostic process is further complicated by overlapping radiographic features between WT and other renal tumors or benign lesions, particularly on preoperative computed tomography (CT) imaging [3]. Although contrast-enhanced CT remains a cornerstone for staging and anatomical assessment, its interpretation is inherently dependent on the radiologist’s expertise and subjective pattern recognition. Enhanced heterogeneity, necrosis, hemorrhage, cystic components, and mass effects on adjacent structures may vary widely across cases, further increasing interpretive complexity.

As a result, small WT lesions may be overlooked or misinterpreted as benign entities, while benign lesions may conversely be suspected as malignant. This challenge is especially pronounced in early-stage or atypical presentations, where subtle imaging findings may not conform to classic textbook descriptions. Distinguishing WT from other renal pathologies and reliably identifying small lesions therefore remain crucial yet challenging aspects of pediatric oncologic care. An objective, reproducible, and high-sensitivity diagnostic support mechanism could meaningfully enhance radiological confidence and reduce variability in interpretation.

In recent years, medical imaging has undergone a significant transformation with the emergence of deep learning (DL), which is a subset of artificial intelligence (AI). DL-based approaches, particularly convolutional neural networks, have demonstrated remarkable potential in extracting high-dimensional imaging features that may not be perceptible to the human eye, thereby improving diagnostic accuracy across a wide range of clinical applications [5]. These models learn hierarchical representations directly from raw image data, enabling automated detection, classification, and segmentation tasks without reliance on handcrafted feature engineering. In particular, DL methods have shown promising results in the evaluation and classification of renal tumors, offering enhanced precision and efficiency compared with conventional image interpretation [6,7]. Prior studies in renal imaging have demonstrated that AI-driven models can differentiate tumor subtypes, identify malignant characteristics, and assist in lesion segmentation with high-performance metrics [6,7].

Despite the growing impact of deep learning (DL) in medical imaging, its application in pediatric renal and retroperitoneal tumor differentiation remains comparatively underdeveloped. Most AI studies in renal oncology have focused predominantly on adult renal cell carcinoma and related adult imaging tasks, whereas pediatric tumors represent a distinct clinical and biological spectrum with different imaging behaviors, treatment pathways, and diagnostic priorities. In particular, the preoperative distinction between Wilms tumor (WT) and neuroblastoma remains a highly relevant clinical challenge. Although both entities may present as large abdominal masses in childhood, their origin, anatomical relationships, treatment algorithms, and surgical implications differ substantially. In daily practice, this distinction is not always straightforward, especially when lesions are large, heterogeneous, atypical in appearance, or partially visualized on individual CT slices. For this reason, AI approaches that merely provide a global image label may be less informative than models that also localize suspicious regions. Lesion-level detection strategies, which more closely resemble the way radiologists review cross-sectional imaging, remain relatively scarce in this specific pediatric context. A further methodological advantage arises when preoperative imaging is paired with postoperative histopathological diagnosis, which provides a robust reference standard for ground-truth assignment. Anchoring model development to histopathology reduces diagnostic ambiguity, improves label reliability, and strengthens the clinical credibility of the resulting framework

Beyond methodological relevance, there is also a clear practical rationale for developing AI-assisted support tools for pediatric oncologic imaging. Interpretation of pediatric abdominal CT may be challenging even in experienced centers, and consistent subspecialty expertise is not always available in routine practice. Increasing imaging volume, time pressure, and variability in radiologist experience may all contribute to diagnostic uncertainty, especially in cases that do not display classic textbook imaging features. In peripheral hospitals or resource-limited settings, access to dedicated pediatric radiology input may be even more constrained, increasing reliance on general radiologic interpretation under less specialized conditions. In this setting, an automated lesion-localization system capable of highlighting suspicious tumor regions and suggesting a differential category may serve as a practical adjunct to image review. Such a system could help standardize assessment, reduce oversight of subtle or partially visualized lesions, and support more structured interpretation of pediatric abdominal masses. Importantly, the potential value of such tools lies not in replacing radiologists, but in complementing expert assessment by increasing consistency, transparency, and workflow efficiency. Localization-aware outputs may also facilitate communication between radiology, pediatric surgery, and pediatric oncology teams by making model decisions visually interpretable rather than purely probabilistic.

In the present study, we sought to develop a clinically interpretable DL framework for the preoperative differentiation of WT and neuroblastoma on contrast-enhanced pediatric abdominal CT. Rather than framing the problem as a purely global image-classification task, we adopted a lesion-level detection approach that identifies suspicious regions and assigns a tumor category at the lesion level. This design was chosen to better align with radiological workflows and to improve interpretability in a clinically meaningful differential diagnostic setting. Using histopathologically confirmed postoperative diagnoses as the reference standard, we aimed to evaluate whether such a localization-aware model could provide reliable within-dataset performance for distinguishing WT from neuroblastoma while also preserving acceptable background suppression in negative images. By focusing on a clinically relevant pediatric differential diagnosis and an interpretable detection framework, we aimed to establish an exploratory AI-based diagnostic support approach that may serve as a foundation for future patient-level, multicenter, and externally validated studies in pediatric oncologic imaging.

## 2. Materials and Methods

### 2.1. Study Design and Objective

This study was designed as a single-center, retrospective, image-level exploratory diagnostic support study to evaluate whether an artificial intelligence-assisted lesion-localization framework could help differentiate Wilms tumor from neuroblastoma on preoperative pediatric abdominal computed tomography (CT) images. The primary objective was to develop and test a deep learning object-detection model capable of localizing tumor regions and distinguishing between Wilms tumor and neuroblastoma on axial CT PNG images. Secondary objectives were to characterize false-positive detections on negative/background images and to generate qualitative examples of correct and incorrect lesion localization for visual error analysis.

The study was intentionally structured as an exploratory imaging analysis rather than a patient-level diagnostic accuracy study. The available dataset consisted of archived PNG exports with lesion annotations, but without consistently recoverable patient-level linkage across all files. Accordingly, all data partitioning, model development, and performance analyses were performed at the image level. The reported performance therefore reflects within-dataset image-level behavior and should not be interpreted as patient-level clinical diagnostic accuracy.

### 2.2. Study Population and Eligibility Criteria

The image dataset was assembled from preoperative abdominal CT examinations of pediatric patients with histopathologically confirmed Wilms tumor or histopathologically confirmed abdominal neuroblastoma. Positive images were derived from examinations in which the tumor was visible and suitable for manual lesion annotation. In addition, negative/background CT images without an annotated target lesion were retained to assess the false-positive behavior of the model during inference.

Eligible images met the following criteria: (1) availability of preoperative abdominal CT imaging in PNG format, (2) sufficient image quality for lesion review and annotation, and (3) definitive postoperative histopathological confirmation of either Wilms tumor or neuroblastoma. Images were excluded if they were corrupted, lacked their corresponding annotation files, contained malformed labels, or had non-standard formatting likely to compromise reliable model input. To preserve geometric consistency in the primary analysis, markedly non-standard images were identified during dataset auditing and treated separately during quality review.

Histopathology served as the reference standard for tumor class assignment. Each positive image inherited the diagnosis of its corresponding lesion as either Wilms tumor or neuroblastoma. Negative/background images contained no annotated target lesion and were represented by empty annotation files. Because reliable patient-level linkage was not consistently preserved in the archival PNG export, no patient-level aggregation was used in the primary analysis.

### 2.3. CT Acquisition Protocol and Radiological Review

All CT examinations were performed as part of routine clinical care using a 128-slice multidetector CT scanner (Siemens Somatom Definition AS, Forchheim, Germany; Syngo CT VA48A). A standardized pediatric abdominal contrast-enhanced CT protocol was used to balance diagnostic image quality with radiation safety. Examinations were acquired at 100 kV with automated tube-current modulation ranging from 30 to 120 mA according to patient size and abdominal circumference, in line with institutional pediatric dose-reduction practice.

Radiological review was performed with support from an experienced pediatric radiologist who routinely interprets pediatric abdominal CT examinations. Lesion location, size, internal morphology, enhancement pattern, anatomic relationships, and overall radiologic conspicuity were assessed before and during annotation. This structured radiologic review ensured consistent lesion identification and reduced the risk of annotation drift. The radiologist also participated in the adjudication of uncertain images in which lesion margins or lesion extent were not straightforward on a single slice.

### 2.4. Image Annotation and Ground-Truth Definition

Tumor-containing images were manually annotated using a bounding-box approach in YOLO-compatible format. Bounding boxes were drawn around all visible tumor regions on axial images judged to contain lesion tissue. Annotation was performed on an image-by-image basis to enable lesion-level object-detection training by an expert pediatric surgeon. Ambiguous images, particularly those with partial lesion visibility, indistinct margins, or uncertainty regarding lesion extent, were jointly reviewed by the annotating investigator and the pediatric radiologist, and only consensus-positive images were retained in the final annotated set.

Two target lesion classes were defined for model development: neuroblastoma and Wilms tumor. In the raw dataset structure these classes were originally labeled as nonwt and wt, respectively; for the purposes of the present study and manuscript, they were recoded conceptually as neuroblastoma and Wilms tumor. Background-negative images were not modeled as a third detection class. Instead, they were preserved with empty label files, consistent with standard object-detection practice in which the detector learns both to localize target objects and to suppress detections in images lacking target lesions.

### 2.5. Dataset Composition and Partitioning

The final image corpus comprised 3553 abdominal CT PNG images. Of these, 2103 were positive lesion-containing images and 1450 were background-negative images represented by empty annotation files. Across all positive images, a total of 2155 annotated lesion instances were available, including 929 neuroblastoma annotations and 1226 Wilms tumor annotations.

The dataset was partitioned at the image level into training, validation, and test subsets using a stratified 70%/20%/10% allocation scheme while preserving the relative proportions of neuroblastoma-positive images, Wilms tumor-positive images, and background-negative images. The final split consisted of 2485 training images, 709 validation images, and 359 test images. The training subset contained 1471 positive images and 1014 background-negative images. The validation subset contained 419 positive images and 290 background-negative images, and the test subset contained 213 positive images and 146 background-negative images.

Because the archival dataset did not allow robust patient-level reconstruction across all exported PNG files, partitioning could not be performed at the patient level. This limitation was recognized before model interpretation and is central to the scope of this study. In addition, a filename-based audit revealed non-random source-family patterns across images, raising the possibility of source-domain confounding. This issue was documented and considered explicitly when interpreting model performance.

**Figure 1:**
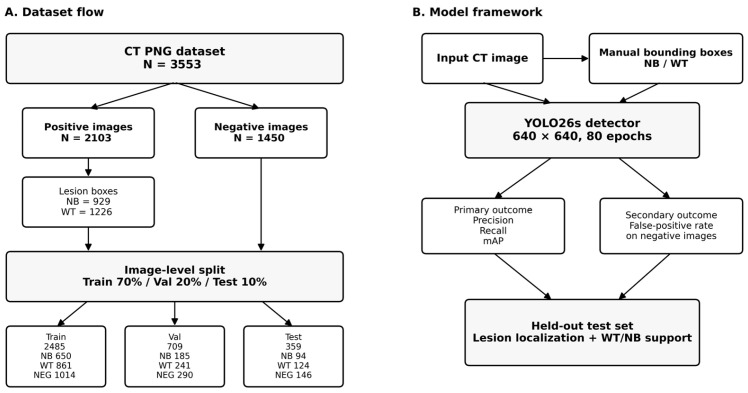
Study flowchart and methodological framework.

The study flowchart and methodological framework can be seen in Figure 1.

### 2.6. Data Auditing, Cleaning, and Preprocessing

Before model training, the dataset underwent structural and quality-control auditing. This audit confirmed one-to-one correspondence between image and label files, valid class identifiers, and syntactically valid YOLO annotation structures in the retained primary dataset. All images were reviewed for input-size consistency. A small number of markedly non-standard low-resolution images were identified during quality control; these were recognized as potential sources of artifactual signals and were considered during preprocessing review and sensitivity planning.

Model input preprocessing was intentionally kept simple in order to preserve reproducibility and avoid introducing undocumented handcrafted transformations. Images were used as full-frame axial PNG inputs, and resizing to the network input resolution was handled by the training framework. No manual lesion cropping, radiomic feature engineering, or custom image normalization pipeline was applied outside the detector’s native preprocessing workflow.

### 2.7. Deep Learning Architecture and Training Strategy

A single contemporary one-stage object detector, YOLO26s, was selected a priori as the prespecified architecture for the primary analysis. The model was implemented using the Ultralytics YOLOv8 framework (Ultralytics version 8.4.31) with PyTorch (v2.1.0) and CUDA (v12.1) acceleration. The choice of a single modern detector was deliberate and reflected the clinical objective of this study: to evaluate a localization-aware framework for a focused differential diagnostic problem rather than to perform broad architecture benchmarking.

Training was initialized from pretrained weights and conducted using a 640 × 640 input resolution, a batch size of 16, mixed-precision training, and 80 training epochs with early stopping patience set to 20 epochs. Built-in augmentation settings within the Ultralytics detection framework were retained to improve robustness to minor spatial variation while avoiding excessive manual augmentation engineering. Model development was performed on a workstation equipped with an NVIDIA GeForce RTX 3090 Ti graphics card (24 GB VRAM) and 32 GB of system memory. The best-performing checkpoint on the validation set was selected for final evaluation on the held-out test subset without further parameter tuning.

### 2.8. Outcome Measures and Statistical Analysis

The primary endpoint was lesion-level detection and class-specific discrimination between Wilms tumor and neuroblastoma on the held-out test set. The principal performance metrics were precision, recall, mean average precision at an intersection-over-union threshold of 0.50 (mAP@0.5), and mean average precision averaged across intersection-over-union thresholds from 0.50 to 0.95 in 0.05 increments (mAP@0.5:0.95), as implemented in the Ultralytics validation pipeline. Precision was defined as the number of true-positive detections divided by the sum of true-positive and false-positive detections, whereas recall was defined as the number of true-positive detections divided by the sum of true-positive and false-negative detections. mAP@0.5:0.95 corresponded to the COCO-style averaged detection metric rather than a single-threshold Pascal VOC-style summary.

Secondary analysis focused on image-level false-positive behavior in the background-negative subset. A negative image was considered a false positive if at least one predicted bounding box exceeded the predefined confidence threshold used for test-time inference. The image-level false-positive rate was calculated as the number of negative images producing at least one false-positive detection divided by the total number of background-negative test images.

Because this was a prespecified single-model exploratory study and not a formal multi-model superiority analysis, statistical inference emphasized transparent effect-size reporting and error characterization rather than *p*-value-driven hypothesis testing. Representative true-positive, false-positive, and false-negative examples were exported for qualitative figure preparation.

### 2.9. Ethical Considerations

This study was approved by the institutional ethics committee of Kocaeli University Faculty of Medicine (KOU GOKAEK IRB). All data were anonymized before annotation and analysis. The requirement for informed consent was waived because of the retrospective design and the use of de-identified archival imaging data. All study procedures were conducted in accordance with institutional ethical standards and the principles of the Declaration of Helsinki.

## 3. Results

The final dataset comprised 3553 CT PNG images. After image-level stratified partitioning, the training set included 2485 images; the validation set contained 709 images; and the test set included 359 images. Across these subsets, the training set contained 650 neuroblastoma and 861 Wilms tumor lesion annotations together with 1014 background-negative images; the validation set contained 185 neuroblastoma and 241 Wilms tumor annotations together with 290 background-negative images; and the test set contained 94 neuroblastoma and 124 Wilms tumor lesion annotations together with 146 background-negative images. At the image level, the held-out test set consisted of 213 positive images and 146 negative/background images (Figure 1 and Table 1).

**Figure 2:**
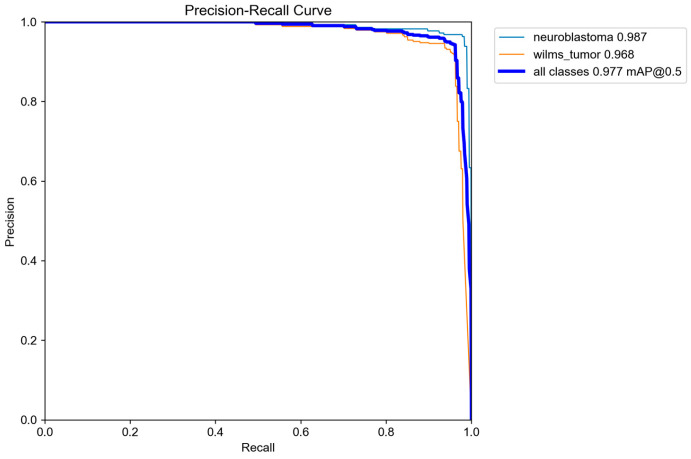
PR curve and mAP scores of the trained model.

During internal validation, the YOLO26s detector achieved an overall precision of 0.948, a recall of 0.964, an mAP@0.5 of 0.977, and an mAP@0.5:0.95 of 0.733. Class-specific validation performance was also high. For neuroblastoma, the model achieved a precision of 0.967, a recall of 0.978, an mAP@0.5 of 0.987, and an mAP@0.5:0.95 of 0.743. For Wilms tumor, the corresponding validation values were a precision of 0.928, a recall of 0.950, an mAP@0.5 of 0.968, and an mAP@0.5:0.95 of 0.723 (Table 2 and Figure 2).

**Figure 3:**
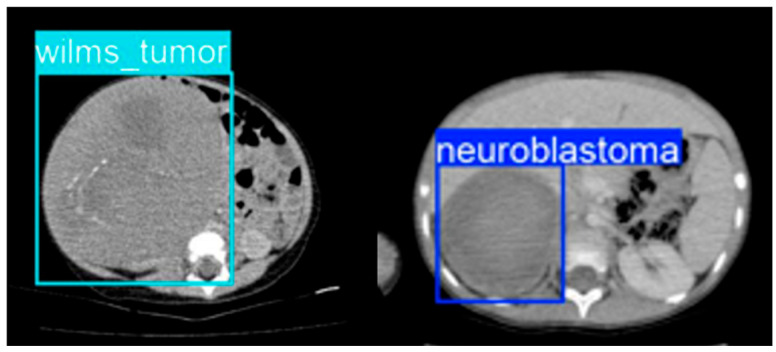
Sample detection results.

On the held-out test set, lesion-level performance remained strong. Overall test-set precision was 0.954, with a recall of 0.951, an mAP@0.5 of 0.977, and an mAP@0.5:0.95 of 0.732. For neuroblastoma, the model achieved a precision of 0.948, a recall of 0.926, an mAP@0.5 of 0.971, and an mAP@0.5:0.95 of 0.734. For Wilms tumor, the corresponding values were a precision of 0.960, a recall of 0.976, an mAP@0.5 of 0.983, and an mAP@0.5:0.95 of 0.730. Thus, lesion localization performance was high for both tumor classes, without a substantial performance drop in either category. Sample detection results can be seen in Figure 3.

In a detector-derived image-level binary analysis comparing tumor-present versus no-tumor/background images, 212 of 213 positive test images were flagged by at least one retained detection, whereas 130 of 146 negative/background images produced no detection. This corresponded to an image-level sensitivity of 99.5%, a specificity of 89.0%, a positive predictive value (PPV) of 93.0%, a negative predictive value (NPV) of 99.2%, and an overall accuracy of 95.3%. The complementary false-positive analysis showed that 16 of 146 negative/background images generated at least one false-positive detection, corresponding to an image-level false-positive rate of 10.96%.

A detector-derived image-level class analysis was also performed for the two tumor categories. Among 94 neuroblastoma-positive images, 93 were labeled as neuroblastoma and one was not retained as a detected lesion. Among 119 Wilms tumor–positive images, all 119 were labeled as Wilms tumor. No direct Wilms-to-neuroblastoma or neuroblastoma-to-Wilms cross-class mislabeling was observed in the retained positive predictions; image-level classification errors arose primarily from a missed detection in one neuroblastoma image and false-positive detections in the background-negative group. Based on this detector-derived image-level output, neuroblastoma showed a sensitivity of 98.9%, a specificity of 98.5%, a PPV of 95.9%, an NPV of 99.6%, and an accuracy of 98.6%. For Wilms tumor, the corresponding values were a sensitivity of 100.0%, a specificity of 95.0%, a PPV of 90.8%, an NPV of 100.0%, and an accuracy of 96.7%. The detector-derived image-level classification report also showed high precision and recall for both tumor classes, with precision/recall/F1 values of 0.9588/0.9894/0.9738 for neuroblastoma and 0.9084/1.0000/0.9520 for Wilms tumor.

## 4. Discussion

In this single-center, retrospective, image-level exploratory study, a localization-aware deep learning detector achieved strong within-dataset performance for differentiating Wilms tumor from neuroblastoma on preoperative pediatric abdominal CT images. On the held-out test set, the model reached a precision of 0.954, a recall of 0.951, an mAP@0.5 of 0.977, and an mAP@0.5:0.95 of 0.732, with consistently high class-specific performance for both neuroblastoma and Wilms tumor. At the image level, the detector also showed very high sensitivity for tumor-present images and a low miss rate while maintaining background suppression in most negative images. These findings support the feasibility of a lesion-localization approach for exploratory diagnostic support in a clinically important pediatric differential diagnostic setting.

Wilms tumor (WT) remains a diagnostically challenging pediatric malignancy due to its heterogeneous imaging appearance and overlap with other renal lesions, particularly in small or early-stage tumors that may be overlooked on conventional computed tomography (CT) imaging [1,2,3,4,8]. Pediatric renal masses encompass a wide spectrum of pathologies with partially overlapping radiological characteristics, and their differentiation often relies on subtle enhancement patterns, the internal architecture, vascular displacement, and the mass effect. In daily clinical practice, such distinctions may be complicated by necrosis, hemorrhage, cystic components, or atypical presentations. Moreover, inter-observer variability in the interpretation of pediatric abdominal CT studies may further contribute to diagnostic inconsistency. Accurate preoperative identification is therefore essential for guiding diagnostic strategies, including biopsy decisions; optimizing imaging workflows; determining surgical planning; and selecting treatment approaches such as neoadjuvant chemotherapy. Even minor diagnostic uncertainty may influence multidisciplinary decision-making and ultimately impact therapeutic sequencing. In this context, improving the reliability and objectivity of imaging-based WT detection represents a clinically relevant and unmet need.

Deep learning (DL) techniques have increasingly transformed medical imaging by enabling automated extraction of complex visual patterns that exceed the capabilities of traditional rule-based systems [5,7,9,10]. Convolutional neural networks (CNNs) are capable of learning hierarchical image representations directly from pixel data, capturing subtle textural and structural information that may not be readily perceptible to the human observer. In renal oncology and other imaging domains, DL-based models have demonstrated high performance in detection, segmentation, and classification tasks, often improving reproducibility and reducing inter-observer variability [5,7,9,10]. Importantly, these systems may standardize interpretation across institutions by providing algorithm-driven consistency. Object-detection frameworks such as YOLO have demonstrated strong performance across multiple medical imaging applications due to their efficiency, real-time processing capability, and precise localization accuracy [11]. Unlike purely classification-based systems, detection models generate spatially explicit outputs in the form of bounding boxes, thereby aligning more closely with clinical radiology workflows. This spatial transparency enhances interpretability and allows clinicians to visually verify algorithm outputs. Building on this paradigm, the present study explored the feasibility of a DL-based lesion detection model for preoperative WT identification using pediatric CT images.

The principal clinical relevance of the present work lies in its focus on Wilms tumor versus neuroblastoma, rather than on Wilms tumor detection against normal controls alone. This distinction is important because the most meaningful preoperative problem in practice is often not whether a mass is simply present, but whether an identified abdominal mass is more likely to represent a renal-origin Wilms tumor or an extra-renal retroperitoneal lesion such as neuroblastoma. These entities differ in biological behavior, treatment planning, surgical implications, and multidisciplinary management pathways. For that reason, an AI framework that attempts to localize the suspicious region and simultaneously assign a lesion class is more clinically relevant than a purely global image classifier that produces only a single image-level label.

Another important strength of this study is its use of a postoperative histopathological diagnosis as the reference standard for lesion class assignment. In oncologic imaging studies, robust ground-truth definition is essential because mislabeled or weakly labeled training data may introduce systematic error and undermine clinical credibility. By anchoring the lesion labels to histopathologically confirmed Wilms tumor and neuroblastoma diagnoses, the present framework reduces diagnostic ambiguity at the class-definition level and improves the methodological rigor of model development. This is particularly important in pediatric tumor imaging, where overlap in radiologic appearance can make image-only diagnostic labeling unreliable if pathology is not used as the reference standard.

The detection-based design also offers a practical interpretability advantage. Rather than providing only an abstract probability score, the model generates lesion-localizing outputs that can be visually inspected. This is more closely aligned with routine radiological workflows, in which suspicious regions are identified spatially and then interpreted in an anatomical and clinical context. In correctly detected cases, the model generally localized the dominant lesion region with class-consistent bounding boxes, suggesting that the detector was not merely producing image-level labels but was identifying spatially meaningful targets. In a clinical support setting, this type of output is more likely to be accepted by readers than a purely opaque classification score.

The secondary analysis of negative/background images provides additional context for potential practical use. Among 146 background-negative test images, 16 produced at least one false-positive detection, corresponding to an image-level false-positive rate of 10.96%. Although the majority of negative images remained free of any retained box, this result also highlights an important limitation of the current framework: strong lesion-level mAP does not eliminate false alarms in non-lesion images. In a real workflow, such false positives may increase unnecessary attention to benign or irrelevant findings, especially if the system is used as a triage-support tool. For this reason, the false-positive behavior should be viewed as an integral part of model performance rather than as a peripheral observation. The present findings should also be interpreted in the context of the current literature. While deep learning has shown substantial promise in medical imaging, much of the renal AI literature has focused on adult renal tumors, especially renal cell carcinoma, rather than pediatric abdominal tumor differentiation. Pediatric tumors differ from adult renal neoplasms not only in histopathology and biology but also in imaging presentation and diagnostic workflow. In addition, many previous AI studies have emphasized image-level classification rather than lesion localization. In this context, the current study contributes to the field by focusing on a pediatric differential diagnosis of genuine clinical relevance and by using a lesion-detection framework that better reflects the way radiologists review cross-sectional imaging.

Several limitations should be emphasized. First, this study was retrospective and single-center, which inherently limits generalizability. Scanner settings, contrast timing, export procedures, and patient mix may vary across institutions, and the present results should not be interpreted as evidence of external robustness. Second, the dataset structure did not permit reliable patient-level linkage across all archival PNG files. Consequently, this study was conducted at the image level rather than at the patient level. This distinction is critical. Although the held-out test performance was strong, it reflects image-level within-dataset behavior and should not be conflated with patient-level diagnostic accuracy. Third the dataset was intentionally restricted to Wilms tumor, neuroblastoma, and background-negative normal kidney images. Although this made the differential diagnostic question more clinically meaningful than a WT-versus-normal design, it still does not capture the full spectrum of pediatric renal and retroperitoneal pathology. Other important mimics, including clear cell sarcoma, rhabdoid tumors, congenital mesoblastic nephroma, inflammatory lesions, and other uncommon abdominal masses, were not represented as explicit target classes. As a result, the present model should not be interpreted as a comprehensive pediatric abdominal tumor classifier. Fifth, no direct head-to-head comparison with pediatric radiologists was performed, and no formal reader study was conducted. Therefore, the current results support technical feasibility and translational promise, but not superiority over expert human interpretation.

Future work should focus on strengthening clinical validity rather than simply expanding model complexity. The most important next steps are external validation across independent institutions, restoration of reliable patient-level grouping, broader inclusion of competing pediatric tumor entities, and prospective testing in realistic workflow settings. In addition, structured reader-assistance studies would be valuable to determine whether lesion-localizing AI outputs improve sensitivity, diagnostic confidence, or reporting consistency when used alongside routine radiological review. Further refinement of false-positive suppression, calibration of detection confidence, and incorporation of uncertainty-aware outputs may also improve clinical usability.

In summary, this study demonstrates that a lesion-localization deep learning framework can achieve strong within-dataset performance for differentiating Wilms tumor from neuroblastoma on preoperative pediatric abdominal CT images. The combination of pathology-anchored labels, explicit lesion localization, and evaluation on negative/background images provides a clinically relevant exploratory foundation. At the same time, the image-level design, single-center data source, and source-pattern bias risk require careful interpretation. The present results should therefore be viewed as promising but preliminary evidence supporting further patient-level, multicenter, and clinically integrated validation.

## Figures and Tables

**Table 1:** Dataset composition by split.

Split	Total İmages	Positive İmages	Background-Negative İmages	Neuroblastoma Lesion İnstances	Wilms Tumor Lesion İnstances
Training	2485	1471	1014	650	861
Validation	709	419	290	185	241
Test	359	213	146	94	124
Total	3553	2103	1450	929	1226

**Table 2:** Lesion-level detection performance of YOLO26s.

Subset	Class	Images	Instances	Precision	Recall	mAP@0.5	mAP@0.5:0.95
Validation	All	709	426	0.948	0.964	0.977	0.733
Validation	Neuroblastoma	185	185	0.967	0.978	0.987	0.743
Validation	Wilms tumor	234	241	0.928	0.950	0.968	0.723
Test	All	359	218	0.954	0.951	0.977	0.732
Test	Neuroblastoma	94	94	0.948	0.926	0.971	0.734
Test	Wilms tumor	119	124	0.960	0.976	0.983	0.730

## Data Availability

The data presented in this study are not publicly available due to ethical and privacy restrictions related to patient confidentiality. The data may be made available by the corresponding author upon reasonable request and with appropriate institutional approvals.

## Abbreviations

The following abbreviations are used in this manuscript:

AI	Artificial Intelligence
CT	Computed Tomography
DL	Deep Learning
F1	F1 Score
IoU	Intersection over Union
IRB	Institutional Review Board
mAP	Mean Average Precision
MRI	Magnetic Resonance Imaging
NPV	Negative Predictive Value
PPV	Positive Predictive Value
PR	Precision–Recall
WT	Wilms Tumor
YOLO	You Only Look Once
